# Quercetin and luteolin are single-digit micromolar inhibitors of the SARS-CoV-2 RNA-dependent RNA polymerase

**DOI:** 10.1038/s41598-022-14664-2

**Published:** 2022-06-22

**Authors:** Federico Munafò, Elisa Donati, Nicoletta Brindani, Giuliana Ottonello, Andrea Armirotti, Marco De Vivo

**Affiliations:** 1 Molecular Modeling and Drug Discovery Lab, Istituto Italiano Di Tecnologia, via Morego 30, 16163 Genoa, Italy; 2 Analytical Chemistry Facility, Istituto Italiano Di Tecnologia, via Morego, 30, 16163 Genoa, Italy

**Keywords:** Drug discovery, Drug screening, Computational chemistry, Molecular dynamics

## Abstract

Severe acute respiratory syndrome coronavirus 2 (SARS-CoV-2) has rapidly become a global health pandemic. Among the viral proteins, RNA-dependent RNA polymerase (RdRp) is responsible for viral genome replication and has emerged as one of the most promising targets for pharmacological intervention against SARS-CoV-2. To this end, we experimentally tested luteolin and quercetin for their ability to inhibit the RdRp enzyme. These two compounds are ancestors of flavonoid natural compounds known for a variety of basal pharmacological activities. Luteolin and quercetin returned a single-digit IC_50_ of 4.6 µM and 6.9 µM, respectively. Then, through dynamic docking simulations, we identified possible binding modes of these compounds to a recently published cryo-EM structure of RdRp. Collectively, these data indicate that these two compounds are a valid starting point for further optimization and development of a new class of RdRp inhibitors to treat SARS-CoV-2 and potentially other viral infections.

## Introduction

The COVID-19 pandemic, caused by the emerging new severe acute respiratory syndrome coronavirus 2 (SARS-CoV-2), is having a tragic impact on humans and also affecting our economy. Thanks to an unprecedented and extensive collaboration between academia, biotech companies, and governments, vaccines have been discovered to combat and contain this pandemic. Despite the vaccination programs, SARS-CoV-2 continues to be a human threat worldwide. In addition, the emergence of virus variants is an additional threat in relation to the spread of COVID-19. It is likely that COVID-19 will remain an endemic disease^1^. Therefore, small molecule drugs to treat SARS-CoV-2 infections are an additional weapon to fight SARS-CoV-2.

The publication of the viral genome sequence revealed that the SARS-CoV-2 genome is closely related to the earlier SARS-CoV (more than 80% sequence identity) and, to a lesser extent, to MERS-CoV viruses^2^. This information has triggered the identification of druggable targets based on what was already known for SARS-CoV and MERS-CoV. In particular, the spike protein, 3-chymotrypsin-like protease (M^pro^), papalin-like cysteine protease (PL^pro^), and the RNA-dependent RNA polymerase (RdRp) have emerged as potential targets for drug discovery campaigns owing to their crucial role in viral entry and host-cell invasion^3–5^. Specifically, the spike protein recognizes the host receptor, facilitating fusion between the viral envelope and the host cell membrane^6^. The protease M^pro^ catalyzes the proteolysis of polyproteins translated from the viral genome. The RdRp enzyme is responsible for the replication of RNA from an RNA template^7,8^. Therefore, RdRp is a nonstructural protein that plays a crucial role in the virus life cycle, acting during the viral replication and transcription processes^3,4,9,10^. Additionally, the absence of a human RdRp counterpart and the high similarity of RdRp within different RNA viruses make this enzyme an attractive target for drug repurposing and development of drugs for COVID-19 and potentially other viral infections^4,10,11^.

Given RdRp’s essential role, a wide array of approved nucleoside and nucleotide analogs have been considered for repurposing as candidates to block RdRp of SARS-CoV-2^12–15^. Among them, remdesivir and favipiravir have reached clinical trials. But despite the promising inhibitory effects of remdesivir and favipiravir, with EC_50_ values of 0.77 µM and 61.88 μM, respectively^16^, clinical trials showed adverse effects and no statistically significant benefits for hospitalized patients^17^. More recently, another nucleoside analog, molnupiravir, has entered clinical trials. Molnupinavir is an orally available and efficacious ribonucleoside analog inhibitor of influenza viruses and, similarly to remdesivir, it has been repurposed against SARS-CoV-2^18^. However, the RdRp complex of coronavirus can excise erroneous mutagenic nucleotides incorporated into viral RNA, thus creating resistance to nucleotide analog drugs^19,20^. Consequently, non-nucleoside inhibitors could have more desirable mechanisms of action that would limit the development of resistance.

In this context, natural products are another source of active compounds with promising antiviral activity. These compounds may serve as a starting point for the development of newer molecular entities with greater efficacy and affinity, and with fewer side effects^21^. Recently some examples of natural products with appreciable inhibition potency against SARS-CoV-2 RdRp include lycorine and corilagin^22–24^. Also, luteolin and quercetin, which are two ancestors of flavonoid compounds, are known for having a range of basal pharmacological activities that, in addition to their well-established anti-inflammatory properties, have demonstrated antiviral properties against picornavirus (RNA virus) and DNA viruses, such as hepatitis B virus, herpes simplex, and adenovirus^25–27^.

**Figure 1:**
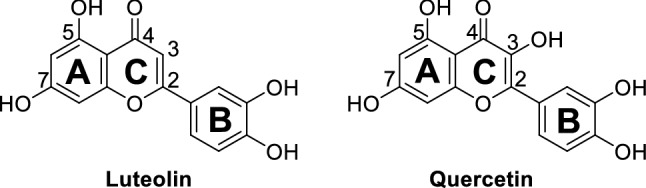
Chemical structures of luteolin and quercetin.

As depicted in Fig. 1, luteolin and quercetin are based on a 15-carbon skeleton with a chromone core comprising bicyclic 1,4-benzopyrone (A- and C-rings) substituted on carbon 2 with a catechol moiety (B-ring). Ring A features a phloroglucinol substitution pattern with two free hydroxyl groups in position 5 and 7. Notably, quercetin differs from luteolin by only one additional hydroxyl group in 3 position.

Luteolin and quercetin have already been the subject of in silico and in vitro studies focused on the SARS-CoV-2 M^pro^ and spike proteins^28,29^. Docking calculations followed by in vitro testing showed that luteolin and quercetin inhibit the viral protease 3CL^pro^ with IC_50_ values in the micromolar range (20 µM and 24 µM, respectively), and with K_i_ ~ 7 μM in the case of quercetin^30–32^. In addition, quercetin was found to be active against two crucial targets of SARS-CoV, namely M^pro^ and NTPase/helicase^33,34^.

Furthermore, molecular docking analysis of natural compounds in the active site of RdRp of SARS-CoV-2 suggest luteolin and quercetin as potential inhibitors of this crucial viral enzyme.[35]Nevertheless, to the best of our knowledge, they have never been experimentally tested on SARS-CoV-2 RdRp. Thus, we decided to evaluate their activity against this specific target. Here, we report their potency and computed binding mode at the viral RdRp target.

## Results and discussion

**Figure 2:**
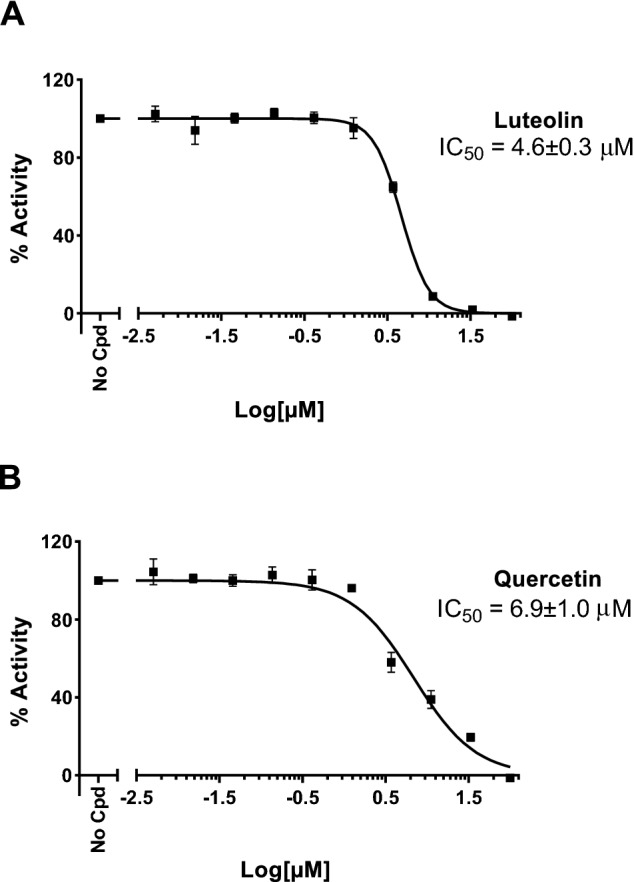
Dose–response curves to calculate IC_50_ values for luteolin (**A**) and quercetin (**B**) by determining each compound’s inhibition activity at 10 different concentrations, ranging from 0.005 µM to 100 µM. The obtained IC_50_ values for luteolin and quercetin are 4.6 ± 0.3 µM and 6.9 ± 1.0 µM, respectively.

First, luteolin and quercetin were tested at two fixed concentrations of 25 µM and 100 µM for their ability to block the viral RdRp target. Both compounds completely inhibited the enzyme at 100 µM, with an inhibition of more than 80% at 25 µM (Table 1). Prompted by these preliminary data, we measured dose–response curves to calculate the IC_50_ values (Table 1) by determining each compound’s inhibition activity at 10 different concentrations, ranging from 0.005 µM to 100 µM (Fig. 2). Luteolin returned an IC_50_ of 4.6 ± 0.3 µM and quercetin an IC_50_ of 6.9 ± 1.0 µM. Thus, both compounds displayed a greater potency against RdRp polymerase than those reported against SARS-CoV-2 M^pro^ and spike proteins (see above).

**Table 1:** In vitro inhibitory activity against SARS-CoV-2 RdRp, kinetic solubility (Sk) in neutral water, microsomal stability (t_1/2_ micr.) in mouse, and plasma stability (t_1/2_ pl.) in mouse of luteolin and quercetin.

Compound	% inhib 100 µM	% inhib 25 µM	IC_50_ (µM)	Sk (µM)	t_1/2_ micr (min)	t_1/2_ pl (min)
Luteolin	100	89	4.6 ± 0.3	21 ± 4	> 60	> 120
Quercetin	100	81	6.9 ± 1.0	16 ± 5	> 60	7 ± 2

Encouraged by such promising single-digit IC_50_ values for these compounds, we decided to evaluate in vitro their drug-like properties, namely aqueous kinetic solubility, together with metabolic and plasma stabilities (Table 1). Luteolin and quercetin have a kinetic solubility of 21 ± 4 µM and 16 ± 5 µM in PBS neutral buffer (pH 7.4), respectively. In terms of metabolic stability, both compounds showed an optimal microsomal stability (t_1/2_ > 60 min). In blood plasma, luteolin was stable in the measured time span (120 min), while quercetin was poorly stable (t_1/2_ = 7 ± 2 min), probably due to the additional hydroxyl group in 3 position.

**Figure 3:**
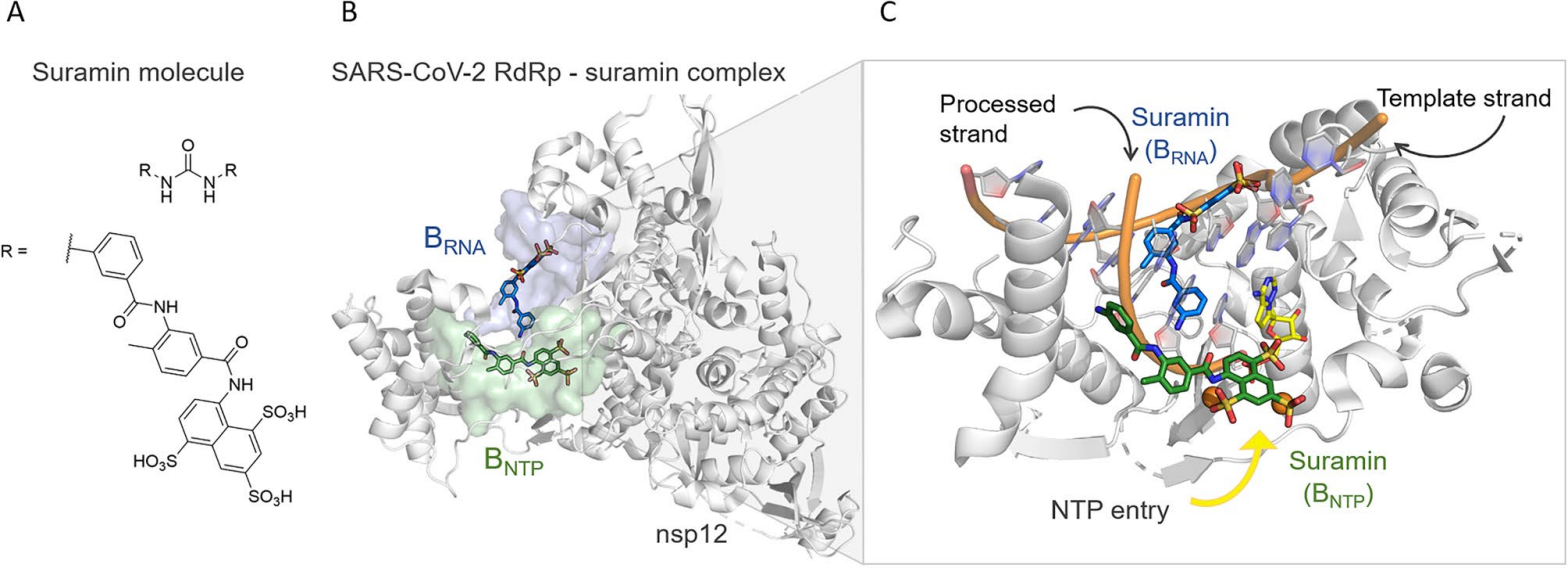
(**A**) 2D structure of suramin. (**B**) Cryo-EM structure (PDB ID 7D4F)^36^ of the RdRp-suramin complex. Only the catalytic nonstructural protein 12 (i.e. nsp12) is depicted. On the left, the two binding pockets with suramin molecules bound, i.e. B_RNA_ and B_NTP,_ are depicted as blue and green surfaces, respectively. (**C**) Close view of the two binding sites, the suramin molecules (as blue and green licorice), the superimposed double-strand RNA (as cartoon), and the incoming nucleotide (as yellow licorice).

A recently published cryoelectron microscopy (cryo-EM) structure of SARS-CoV-2 RdRp in complex with two molecules of suramin (PDB ID 7D4F)^36^, a century-old non-nucleotide analog drug, has revealed two new druggable pockets at the protein target. The binding of suramin to one pocket, B_RNA_ (Fig. 3), prevents the binding of the RNA template strand, while the binding of suramin to the other pocket, B_NTP_ (Fig. 3), prevents both the entry of the nucleotide triphosphate into the catalytic site and the binding of the RNA processed strand (Fig. 3). However, despite a promising IC_50_ value of 0.26 µM^36^, suramin is associated with a high risk of off-target effects on other enzymes in the cell, together with its highly negative charge, which may hinder its penetration into cells^36^. Nevertheless, these two newly identified binding sites at RdRp are suitable pockets to target with non-nucleotide analog drug hits.

Based on these structural findings, we investigated the possible binding modes and the protein–ligand interactions for luteolin and quercetin at the B_RNA_ and B_NTP_ pockets in RdRp. Specifically, the B_RNA_ cavity is formed by the conserved G motif and the N terminus of B motif of the enzyme, and the key residues interacting with suramin are Asn497, Lys500, Arg569, Gln573 and Lys577. In contrast, the B_NTP_ cavity is located near the catalytic active site, which is formed by conserved A, C, E, and F motifs. Here, the key interactions are formed between suramin and Lys551, Arg553, Arg555, Arg836, and Asp865 residues^36^.

**Figure 4:**
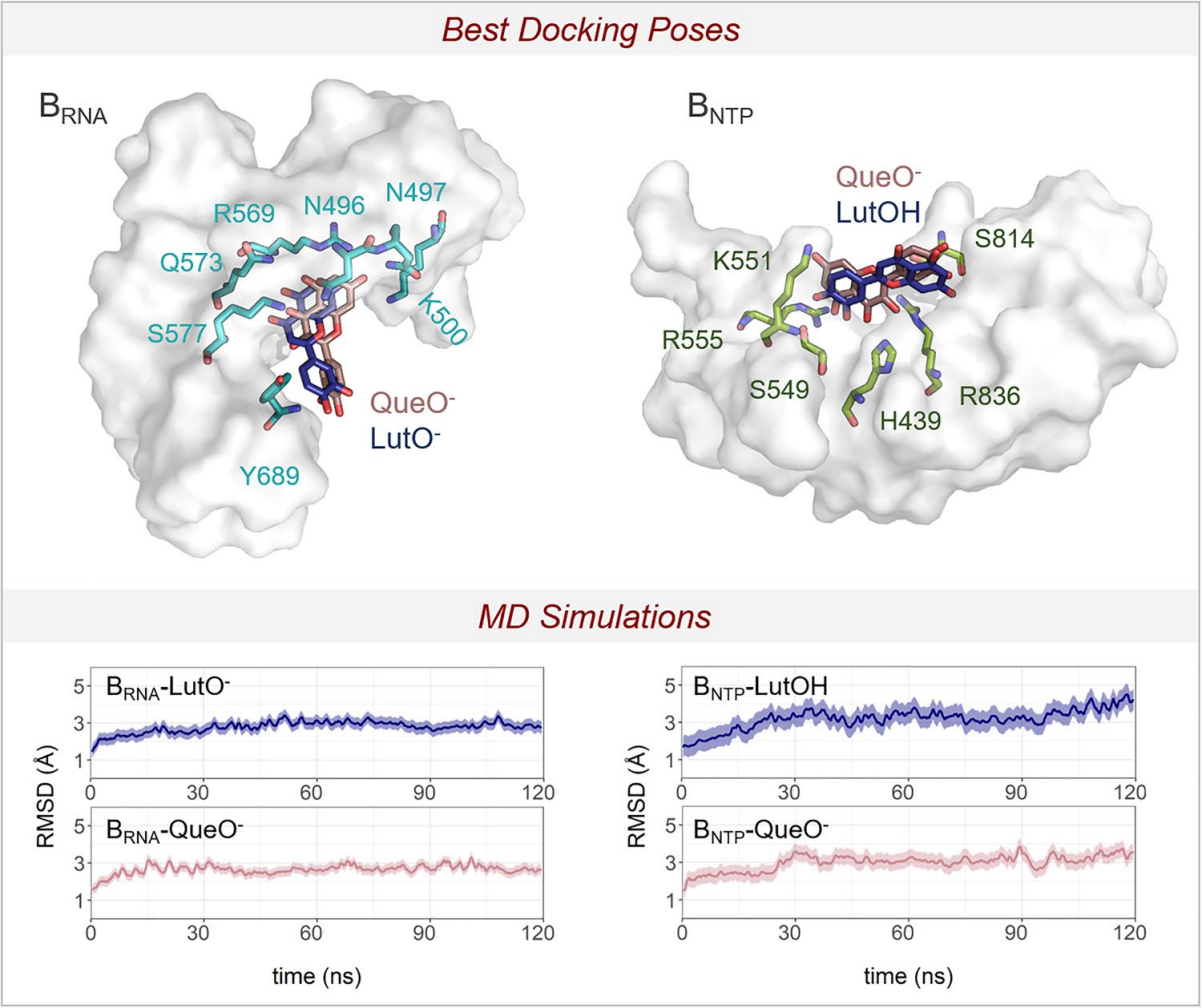
Top: XP Glide docking poses for luteolin and quercetin in dark blue and pink licorice, respectively. The interacting residues are in green licorice for B_NTP_ pocket and light blue for B_RNA_ pocket. Bottom: the root mean squared deviations (RMSD) of the MD simulations for the four systems: i.e. (i) B_RNA_-LutO^-^, (ii) B_RNA_-QueO^-^, (iii) B_NTP_-LutOH, and (iv) B_NTP_-QueO^-^.

To explore the binding modes for luteolin and quercetin, we performed molecular docking of both molecules on the two binding sites (i.e. B_RNA_ and B_NTP_) after removing the suramin (Fig. 4). The Schrödinger's Protein Preparation Wizard tool was used to prepare the protein, with the addition of hydrogens and the prediction of p*K*a values for ionizable residues. Subsequently, an extensive visual inspection was carried to check the overall quality of the final structures. Then, for the molecular docking, luteolin and quercetin were processed with the LigPrep tool^37^ to properly prepare the ligands (e.g. assigning atom charges, converting 2D to 3D structures, and generating tautomeric and ionization states–at pH = 7.0 ± 0.4).

LigPrep generated four structures (i.e. two for luteolin and two for quercetin), which differ in the protonation state of the OH group at position 7 (Fig. S1), namely LutOH, LutO^-^, QueOH, and QueO^-^. These structures were used for protein−ligand docking with Glide. The docking grid was centered on the suramin’s center of mass, either bound to the B_RNA_ or the B_NTP_ pocket. Extra-precision Glide (XP)^38^ was used and a maximum of 20 poses for each molecule were generated (for a total of 24 and 39 poses for B_NTP_ and B_RNA_ pockets, respectively). The resulting docking scores for luteolin and quercetin are shown in the Supporting Information (Table S1). For the B_NTP_ binding pocket, the best docking scores (in kcal mol^-1^) correspond to –7.62 and –5.23, for QueO^-^ and LutOH molecules, respectively. At the B_NTP_ pocket, the main interactions are formed between QueO^-^/LutOH molecules and His439, Ser549, Lys551, Arg555, Ser814, His816, and Arg836 residues (Fig. 4). In contrast, at the B_RNA_ binding pocket, the predicted higher docking scores corresponded to –7.69 and –6.18, for QueO^-^ and LutO^-^ molecules, respectively. The main interactions identified by the molecular docking are between the ligands and Asn496, Asn497, Lys500, Arg 569, Gln573, Lys577, and Tyr689 (Fig. 4).

To further check the stability of the docked structures, we ran equilibrium force-field-based MD simulations (~ 480 ns in total) of the four selected XP poses for luteolin and quercetin at the B_RNA_ and B_NTP_ binding pockets. The integration of experimental results with molecular docking and MD simulations provided a detailed molecular understanding of the inhibitory action of luteolin and quercetin on RdRp. This strategy had already been successfully applied in several other cases^39–43^. A total of four MD simulations of 120 ns each were performed, i.e. (i) B_RNA_-LutO^-^, (ii) B_RNA_-QueO^-^, (iii) B_NTP_-LutOH, and (iv) B_NTP_-QueO^-^ (see Supporting Information for details). In both B_NTP_-LutOH/QueO^-^ systems, the overall ligand-enzyme complex stably maintains the interactions of the starting docking structure. The RMSD mean values for the heavy atoms of the complexes are 3.2 ± 0.5 Å and 3.0 ± 0.4 Å, respectively (Fig. 4). During the MD simulations of B_NTP_-LutOH system, the ligand showed some flexibility over time, with the RMSD mean value for the heavy atoms of 7.5 ± 1.3 Å (Fig. S2). This reflects the reorientation and reorganization of the interactions established between LutOH and the enzyme. Specifically, after the first ~ 50 ns, the ligand moved closer to the side chain of Ser814, Arg836, and Asp865, forming a stable network of interactions that was maintained during the simulations (Fig. S2). Interestingly, these residues also interact through H-bond with suramin in the crystal structure. Additionally, other crystal structures of the SARS-CoV-2 RdRp-RNA complex showed that Ser814 and Arg836 interact with the RNA primer strand^3,44–50^, further supporting the relevance of these residues for ligand recognition and binding. In contrast, B_NTP_-QueO^-^ system showed a slightly more stable conformation of the ligand within the pocket, with an RMSD value for the heavy atoms of 6.3 ± 0.9 Å (Fig. S3). Here, the interactions formed by QueO^-^ with the RdRp enzyme involved His439, Ser549, Ser814, Arg836 side chains (Fig. S3) and the Ile548 backbone. Although His439, Ile548, and Ser549 do not directly participate in RNA binding, they are positioned within ~ 10 Å from the double-strand RNA and from the entry path of the incoming nucleotide.

For the B_RNA_-LutO^-^/QueO^-^ systems, the overall ligand/enzyme complex showed no major differences in the overall stability, as reported by an RMSD mean value for the heavy atoms of 2.8 ± 0.3 Å and 2.6 ± 0.3 Å, respectively (Fig. 4). Here, both ligands showed a reduced flexibility compared to the B_NTP_-LutOH/QueO^-^ systems, with a remarkable stability of the Que-O^-^ ligand at the pocket. Indeed, the RMSD mean values for the heavy atoms of LutO^-^/QueO^-^ are 6.4 ± 2.1 Å and 2.0 ± 0.8 Å, respectively (Figs. S4 and S5). This also reflects the stable H-bond interactions formed by QueO^-^ and RdRp enzyme. In detail, interactions are established between the side chains of Arg569, Gln573, and both LutO^-^ and QueO^-^ ligands (Figs. S4 and S5), while only the latter interacts with the side chain of Asn497, Tyr689, and Ser759 (Fig. S5). Notably, considerable structural evidence (e.g. PDB 6XEZ, 7B3B, 7B3C, 7B3D)^49,50^ shows that these three residues interact with the template strand, thus stabilizing the RNA substrate binding^3,44–50^. Moreover, Ser759 side chain is located close to the incoming nucleotide binding side^51^. These residues are therefore an optimal anchor point for inhibitors of the catalytic activity of the RdRp enzyme.

Overall, the results of our MD simulations indicate that both binding pockets may properly bind and stably host luteolin and quercetin. Nevertheless, the increased stability and higher number of contacts between the B_RNA_ pocket and these ligands suggests that this binding site may be more suitable for ligand binding and structure-based drug design.

These computational insights will serve to start future campaigns for hit-to-lead design, as witnessed recently in computational studies used to guide experiments for drug design targeting viral proteins^52,53^. Notably, Jorgensen et al.^54^ recently performed a virtual screening of ∼2000 approved drugs with a consensus virtual screening protocol used together with MD simulations and biochemical assay. This indicated 14 known drugs active in the micromolar range against 3CL^pro^. Starting from this evidence, the group subsequently applied free-energy perturbation (FEP) calculations to finetune the drug-target interaction of the initial hit, perampanel. This led to the design of a new set of compounds with IC_50_ values in the low nanomolar range, whose binding poses have been corroborated by co-crystal structures^55^. With this successful example in mind, our results now form the basis for a hit-to-lead campaign targeting the SARS-CoV-2 RdRp enzyme.

## Conclusions

In summary, starting from the pharmacological properties of flavonoids, we experimentally tested luteolin and quercetin against SARS-CoV-2 RdRp, a crucial target of the virus responsible for the COVID-19 pandemic. The IC_50_ value in biochemical enzymatic assay is 4.6 ± 0.3 µM for luteolin and 6.9 ± 1.0 µM for quercetin. To the best of our knowledge, this is the first study that quantifies the inhibitory potency of luteolin and quercetin against RdRp, with the evidence of a one-digit micromolar range. Notably, this inhibitory activity is better than previous IC_50_ values reported for these two compounds against other viral proteins of SARS-CoV-2, as M^pro^ and 3CL^pro^. We also investigated and proposed potential binding modes of these compounds to the target protein. Thus, our experimental and computational results complete previous computational investigations that proposed these two known natural products against COVID, providing experimental values for activity and new mechanistic insights^35^. Taken together, our results endorse a further exploration of a new chromone class of RdRp polymerase inhibitors to treat Sars-CoV-2 and potentially other viral infections.

## Methods

### Biochemical assay

The natural flavonoids luteolin and quercetin were tested against SARS-CoV-2 RdRp with an in vitro enzymatic inhibition assay in collaboration with BPS Bioscience. The assay was performed with compounds obtained from commercial sources (luteolin from Fluorochem, quercetin from Sigma-Aldrich). Compounds purity is > 99% based on our HPLC analysis (see SI). The RdRp reactions were conducted in duplicate at 37 °C for 60 min in a 10 µl mixture containing assay buffer (20 mM Tris pH8.0 and 0.01% Triton X100), RNA duplex, ATP substrate and enzyme, and the test compound. The enzyme was produced by BPS Bioscience, and was formulated as 45 mM Tris–HCl pH 8.0, 124 mM NaCl, 2.4 mM KCl, 4 mM MgCl_2_, 1 mM TCEP, 10% glycerol. Typical purity was 95–97%, and typical concentration was 1 mg/ml. These 10 µl reactions were carried out in wells of 384-well Optiplate (PerkinElmer). A 10 mM stock solution of test compound in DMSO was prepared. Dilutions of this stock solution were prepared in assay buffer (5% DMSO concentration) and 2 µl of the dilution was added to a 6 µl of RdRp (final concentration 0.08 mg/mL) containing RNAse inhibitor for preincubation (30 min at room temperature with slow shaking). Reaction was started by addition of 2 µl of the substrate mix containing RNA duplex (40 nM) and ATP substrate (3 µM). Final concentration of DMSO was 1% in all reactions (reference compound–0% DMSO). After enzymatic reactions, 10 µl of anti-Dig Acceptor beads (PerkinElmer, diluted 1:500 with 1 × detection buffer) were added to the reaction mix. After brief shaking, plate was incubated for 30 min. Finally, 10 µl of AlphaScreen Streptavidin-conjugated donor beads (Perkin, diluted 1:125 with 1 × detection buffer) were added. In 30 min, the samples were measured in AlphaScreen microplate reader (EnSpire Alpha 2390 Multilabel Reader, PerkinElmer). In the absence of the compound, the intensity (C_e_) in each data set was defined as 100% of activity. In the absence of the enzyme, the intensity (C_0_) in each data set was defined as 0% of activity. The percent activity in the presence of each compound was calculated according to the following equation: % activity = (C–C_0_)/(C_e_–C_0_), where C is the intensity in the presence of the compound. As a positive control, the reference compound 6-chloropurine-ribose TP was tested at three different concentrations (0.02 µM, 0.2 µM, and 2 µM).

### In vitro microsomial stability

10 mM DMSO stock solution of test compound was pre-incubated at 37 °C for 15 min with mouse liver microsomes added 0.1 M Tris–HCl buffer (pH 7.4). The final concentration was 4.6 µM. After pre-incubation, the co-factors (NADPH, G6P, G6PDH and MgCl_2_ pre-dissolved in 0.1 M Tris–HCl) were added to the incubation mixture and the incubation was continued at 37 °C for 1 h. At each time point (0, 5, 15, 30, 60 min), 30 µL of incubation mixture was diluted with 200 µL cold CH_3_CN spiked with 200 nM of internal standard, followed by centrifugation at 3500 g for 15 min. The supernatant was further diluted with H_2_O (1:1) for analysis. The concentration of test compound was quantified by LC/MS–MS on a Waters ACQUITY UPLC/MS TQD system consisting of a TQD (Triple Quadrupole Detector) Mass Spectrometer equipped with an Electrospray Ionization interface. The analyses were run on an ACQUITY UPLC BEH C18 (50 × 2.1mmID, particle size 1.7 µm) with a VanGuard BEH C18 pre-column (5 × 2.1mmID, particle size 1.7 µm) at 40 °C, using 0.1% HCOOH in H_2_O (A) and 0.1% HCOOH in CH_3_CN (B) as mobile phase. Electrospray ionization (ESI) was applied in positive mode. The percentage of test compound remaining at each time point relative to t = 0 was calculated. The half-lives (t_½_) were determined by an one-phase decay equation using a non-linear regression of compound concentration versus time.

### In vitro plasma stability

10 mM DMSO stock solution of test compound was diluted 50-fold with DMSO-H_2_O (1:1) and incubated at 37 °C for 2 h with mouse plasma added 5% DMSO (pre-heated at 37 °C for 10 min). The final concentration was 2 µM. At each time point (0, 5, 15, 30, 60, 120 min), 50 µL of incubation mixture was diluted with 200 µL cold CH_3_CN spiked with 200 nM of internal standard, followed by centrifugation at 3500 g for 20 min. The supernatant was further diluted with H_2_O (1:1) for analysis. The concentration of test compound was quantified by LC/MS–MS on a Waters ACQUITY UPLC/MS TQD system consisting of a TQD (Triple Quadrupole Detector) Mass Spectrometer equipped with an Electrospray Ionization interface. The analyses were run on an ACQUITY UPLC BEH C18 (50 × 2.1mmID, particle size 1.7 µm) with a VanGuard BEH C18 precolumn (5 × 2.1mmID, particle size 1.7 µm) at 40 °C, using 0.1% HCOOH in H_2_O (A) and 0.1% HCOOH in CH_3_CN (B) as mobile phase. Electrospray ionization (ESI) was applied in positive mode. The response factors, calculated on the basis of the internal standard peak area, were plotted over time. When possible, response vs. time profiles were fitted with Prism (GraphPad Software, Inc., USA) to estimate compounds half-life in plasma.

### Aqueous kinetic solubility

The aqueous kinetic solubility was determined from a 10 mM DMSO stock solution of test compound in Phosphate Buffered Saline (PBS) at pH 7.4. The study was performed by incubation of an aliquot of 10 mM DMSO stock solution in PBS (pH 7.4) at a target concentration of 250 µM resulting in a final concentration of 2.5% DMSO. The incubation was carried out under shaking at 25 °C for 24 h followed by centrifugation at 21.100 g for 30 min. The supernatant was analyzed by UPLC/MS for the quantification of dissolved compound by UV at a specific wavelength (215 nm). The analyses were performed on a Waters ACQUITY UPLC/MS SQD system consisting of a SQD (Single Quadrupole Detector) Mass Spectrometer equipped with Electrospray Ionization interface. The analyses were run on an ACQUITY UPLC BEH C18 column (50 × 2.1mmID, particle size 1.7 µm) with a VanGuard BEH C18 pre-column (5 × 2.1mmID, particle size 1.7 µm), using 10 mM NH_4_OAc in H_2_O at pH 5 adjusted with AcOH (A) and 10 mM NH_4_OAc in CH_3_CN-H_2_O (95:5) at pH 5 (B) as mobile phase.

### Molecular docking of luteolin and quercetin with SARS-CoV-2 RdRp

First, the SARS-CoV-2 RdRp was retrieved from PDB database (PDB ID 7D4F^1^) and subsequently prepared for docking. The preparation was carried by Schrödinger’s Protein Preparation Wizard tool and included: (i) addition of hydrogen atoms, (ii) elimination of water molecules not involved in ligand-binding interaction, (iii) assignment of atomic charges. Subsequently, energy minimized 3D molecular structures of luteolin and quercetin were generated and prepared for docking using LigPrep tool^37^. Additionally, possible ionization states were generated using LigPrep tool^37^, thus resulting in tow possible states for each molecules (see Fig. S1). Eventually, the SARS-CoV-2 RdRp structure (PDB ID 7D4F^36^) was used for docking luteolin and quercetin. The grid was centered on the suramin’s center of mass, and the docking was performed using Glide XP methodology^38,56^.

### Structural models for molecular dynamics simulations

We used four different systems for the MD simulations: (i) B_RNA_-LutO^-^, (ii) B_RNA_-QueO^-^, (iii) B_NTP_-LutOH and (iv) B_NTP_-QueO^-^. Each system was solvated with a 12-Å layer of TIP3P water molecules, and Na^+^ ions were added to neutralize the net charge of the systems. The final models include a total of ~ 183,000 atoms.

### Molecular dynamics simulations set-up

We used force-field-based MD simulations to check the stability of the docked structures. Here, the AMBER-ff14SB^57^ force field was used to treat the RdRp enzyme. All four ligands were parametrized with the general Amber force field (GAFF)^58^, and the atomic charges were derived using the RESP procedure, according to the Merz − Singh − Kollman scheme^59^. Na^+^ metal ions were treated using the Joung–Cheatham parameters^60^. A time integration step of 2 fs was used and the lengths of all bonds involving hydrogen atoms were constrained using the P-LINCS algorithm^61^. A velocity-rescaling thermostat was used to set a system temperature of 310 K^62^, while the Parrinello–Rahman barostat maintained a constant pressure of 1 bar^63^. Long-range electrostatic interactions were calculated with the particle mesh Ewald (PME) method using a Fourier grid spacing of 1.6 Å. Periodic boundary conditions in the three directions of Cartesian space were applied. All MD simulations were performed with Amber2020. The systems were all subject to the same MD simulations procedure. First, we carried out energy minimization to relax the water molecule and the ions. Here, both the ligand and the RdRp backbone were kept fixed with harmonic positional restraints of 300 kcal mol^-1^ Å^2^. Then, the systems were heated up from 0 to 310 K with NVT simulations for a total of 1 ns with 300 kcal mol^-1^ Å^2^ restraints on the ligand. Additionally, 1 ns of simulations in NPT ensemble was performed with the same positional restraints used in the NVT simulations. Three additional NPT simulations of 1 ns each were performed gradually removing the restraints on the ligand. Finally, a production run were performed in the NPT ensemble for each system. We collected overall ~ 480 ns of MD trajectories, specifically ~ 120 ns for each system.

## Supplementary Information

Supplementary Information.
