# Supplementary material for: Quercetin and luteolin are single-digit micromolar inhibitors of the SARS-CoV-2 RNA-dependent RNA polymerase
Source: Sci Rep. 2022 Jun 22;12:10571. doi: 10.1038/s41598-022-14664-2 (PMC9216299; doi:10.1038/s41598-022-14664-2)
Supplement: Supplementary file 1 — Supplementary Information. [file 41598_2022_14664_MOESM1_ESM.pdf]

SUPPORTING INFORMATION

Quercetin and Luteolin are single-digit micromolar inhibitors of the SARS- CoV-2 RNA-dependent RNA polymerase

Federico Munafò,[1,#] Elisa Donati,[1,#] Nicoletta Brindani,[1] Giuliana Ottonello,[2]

Andrea Armirotti[2], Marco De Vivo[1,*]

1. Molecular Modeling and Drug Discovery Lab, Istituto Italiano di Tecnologia, via Morego 30, 16163 Genova, Italy
2. Analytical Chemistry Facility, Istituto Italiano di Tecnologia, via Morego, 30, 16163 Genoa, Italy

Corresponding authors:
Marco De Vivo E-mail: marco.devivo@iit.it

#Equally contributed.

Table of contents

- Chromatography analysis of luteolin and quercetin S2
- Computational results – detail S4

1. Chromatography analysis of luteolin and quercetin

The chromatographic analyses were run on an ACQUITY UPLC BEH C18 (50x2.1mmID, particle size 1.7µm) with a VanGuard BEH C18 pre-column (5x2.1mmID, particle size 1.7µm) (LogD>1). The mobile phase was 10mM NH_4OAc in H_2O at pH 5 adjusted with AcOH (A) and 10mM NH_4OAc in CH_3CN-H_2O (95:5) at pH 5 (B). The mobile-phase B proportion increased from 10 % to 90 % in 7 min.

Luteolin

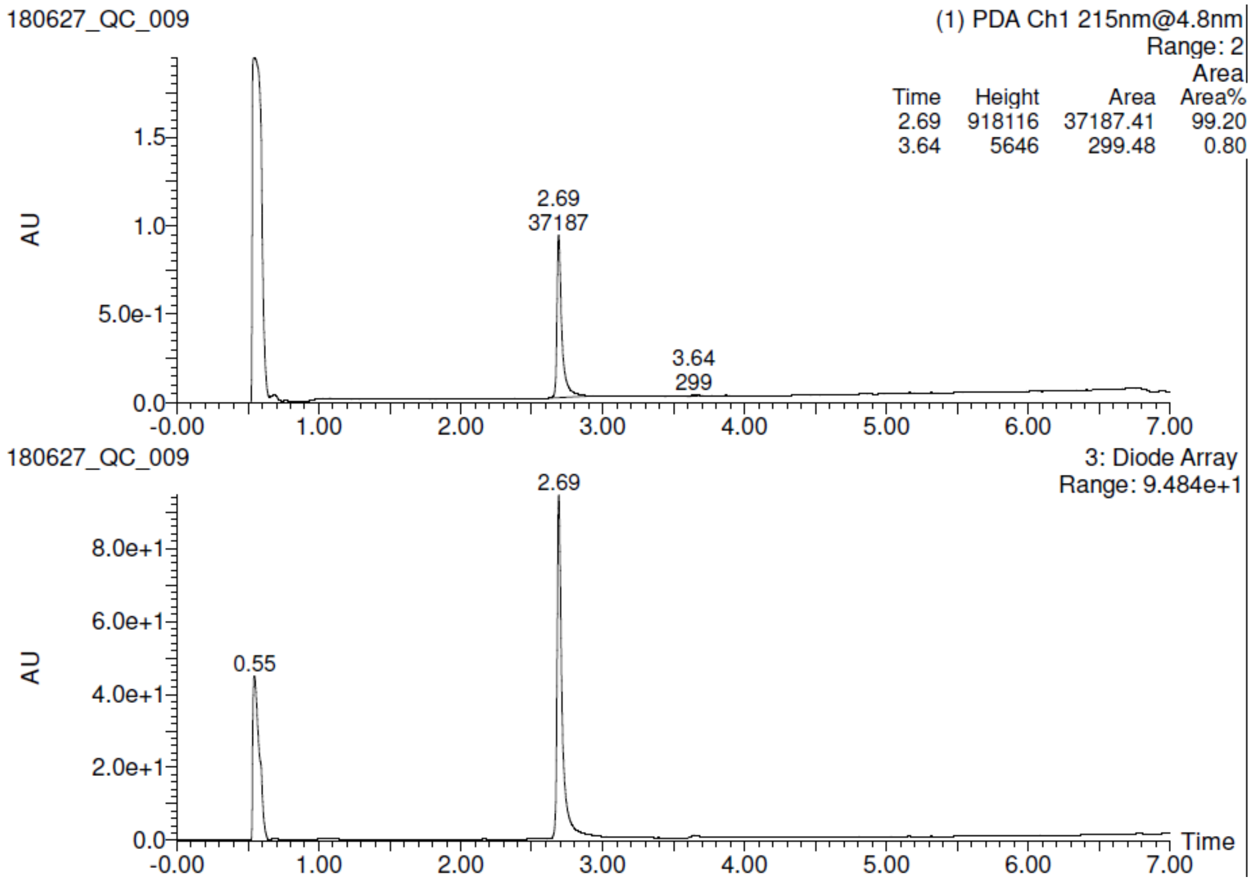

Quercetin

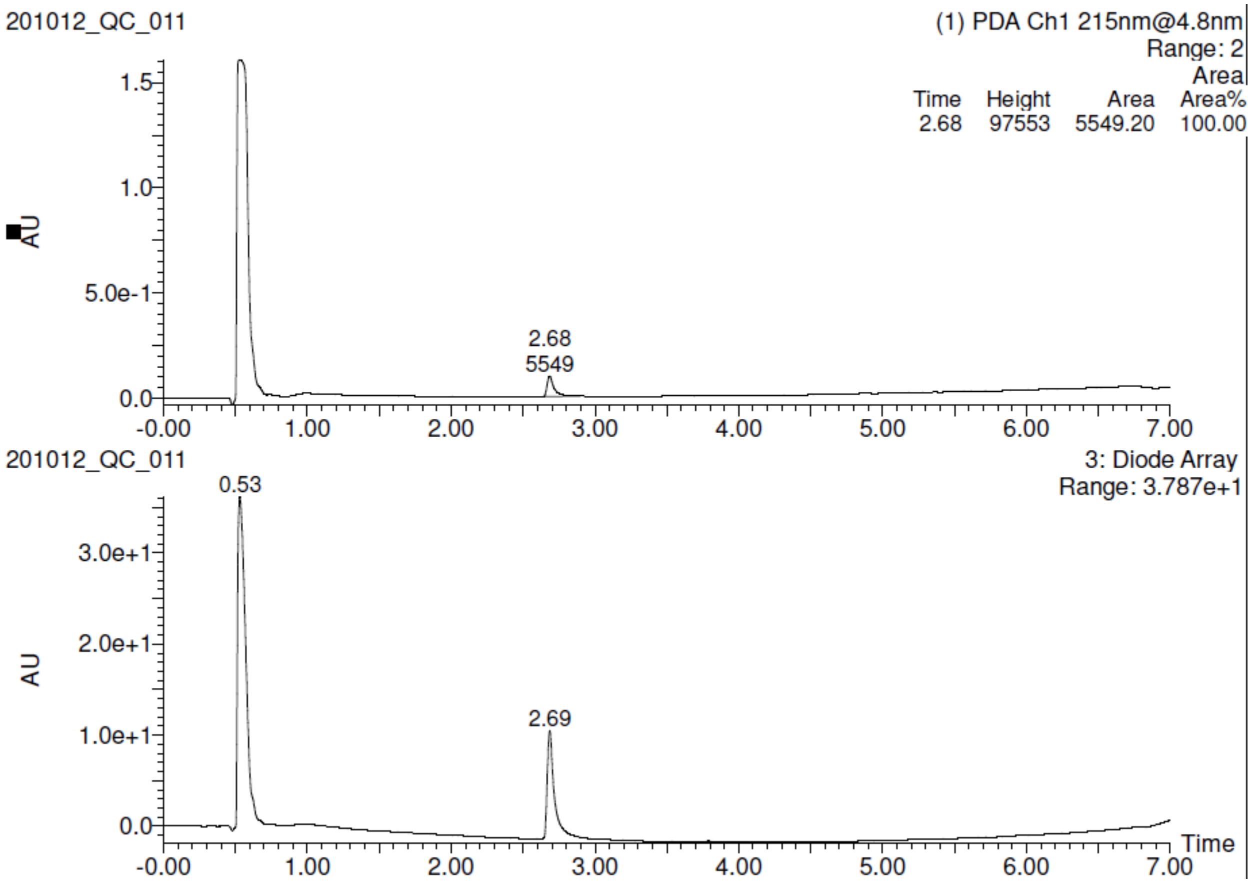

201012_QC_011
(1) PDA Ch1 215nm@4.8nm
Range: 2
Area
Time Height Area Area%
2.68 97553 5549.20 100.00
AU
1.5
1.0
5.0e-1
0.0
2.68
5549
-0.00 1.00 2.00 3.00 4.00 5.00 6.00 7.00
201012_QC_011
3: Diode Array
Range: 3.787e+1
0.53
3.0e+1
2.0e+1
1.0e+1
0.0
2.69
Time

2. Computational studies - Detail

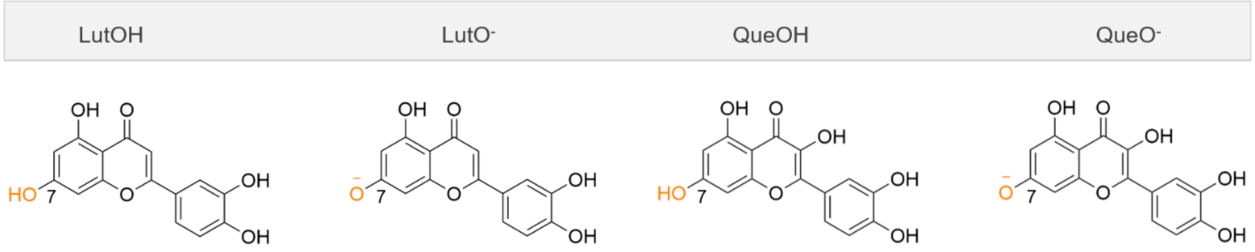

Figure S1. Chemical structures of luteolin and quercetin in different protonation states.

Table S1. Docking scores of both ligands in each protonation state, obtained from XP Glide for both binding pockets (i.e. B_{RNA} and B_{NTP}).

B_{RNA} binding pocket		B_{NTP} binding pocket	
**Name**	**GScore**	**Name**	**GScore**
*QueO⁻	-7,689	*QueO⁻	-7,62
QueO⁻	-7,656	QueOH	-5,443
QueO⁻	-7,541	*LutOH	-5,228
QueO⁻	-7,25	LutOH	-5,172
QueO⁻	-7,191	LutOH	-4,746
QueO⁻	-7,168	QueOH	-4,534
QueO⁻	-6,554	QueO⁻	-4,309
*LutO⁻	-6,175	QueO⁻	-4,199
LutO⁻	-6,122	QueOH	-4,178
LutO⁻	-6,081	QueOH	-4,098
QueOH	-5,977	QueOH	-4,076
QueOH	-5,939	QueO⁻	-4,054
QueOH	-5,935	QueO⁻	-3,807
QueOH	-5,927	LutO⁻	-3,464
QueOH	-5,923	QueO⁻	-3,354
LutO⁻	-5,869	QueOH	-3,351
QueOH	-5,801	QueO⁻	-3,209
QueOH	-5,722	LutOH	-3,181
QueOH	-5,48	LutOH	-3,077
QueOH	-5,446	LutOH	-3,074
QueOH	-5,439	LutO⁻	-3,039
LutO⁻	-5,21	LutOH	-2,902
LutO⁻	-5,187	LutOH	-2,889
LutO⁻	-5,161	LutOH	-2,864
LutO⁻	-5,111		
LutO⁻	-5,028		
LutO⁻	-4,99		
LutO⁻	-4,899		
LutO⁻	-4,88		
LutO⁻	-4,603		
LutO⁻	-4,34		
LutO⁻	-4,148		
QueO⁻	-3,95		
QueO⁻	-3,828		
LutOH	-3,501		
LutOH	-3,009		
LutOH	-2,945		
LutOH	-2,89		
LutO⁻	-2,469		

*in red selected poses for subsequent MD simulations

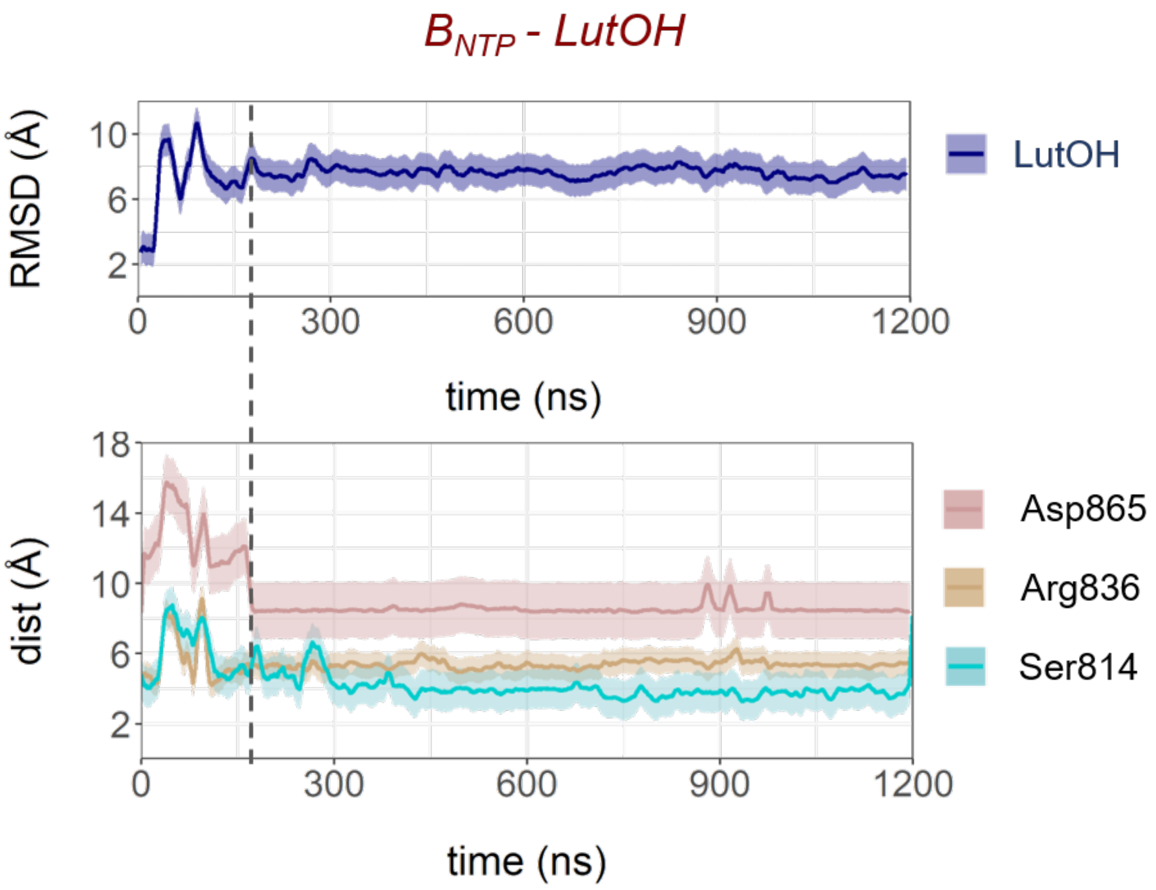

Figure S2. MD simulation of the B_{NTP}-LutOH system. (Top) Time evolution of the root mean square deviation (RMSD) for the heavy atoms of LutOH. (Bottom) Time evolution of the distances between the center of mass (C.O.M.) of the heavy atoms of the LutOH ligand and i) the C.O.M. of the heavy atoms of the guanidinium group of Arg836 (in light brown), ii) the C.O.M. of the heavy atoms of the carboxyl group of Asp865 (in pink), and iii) the C.O.M. of the heavy atoms of the side chain of Ser814 (in cyan). The dashed line marks the reorganization of LutOH.

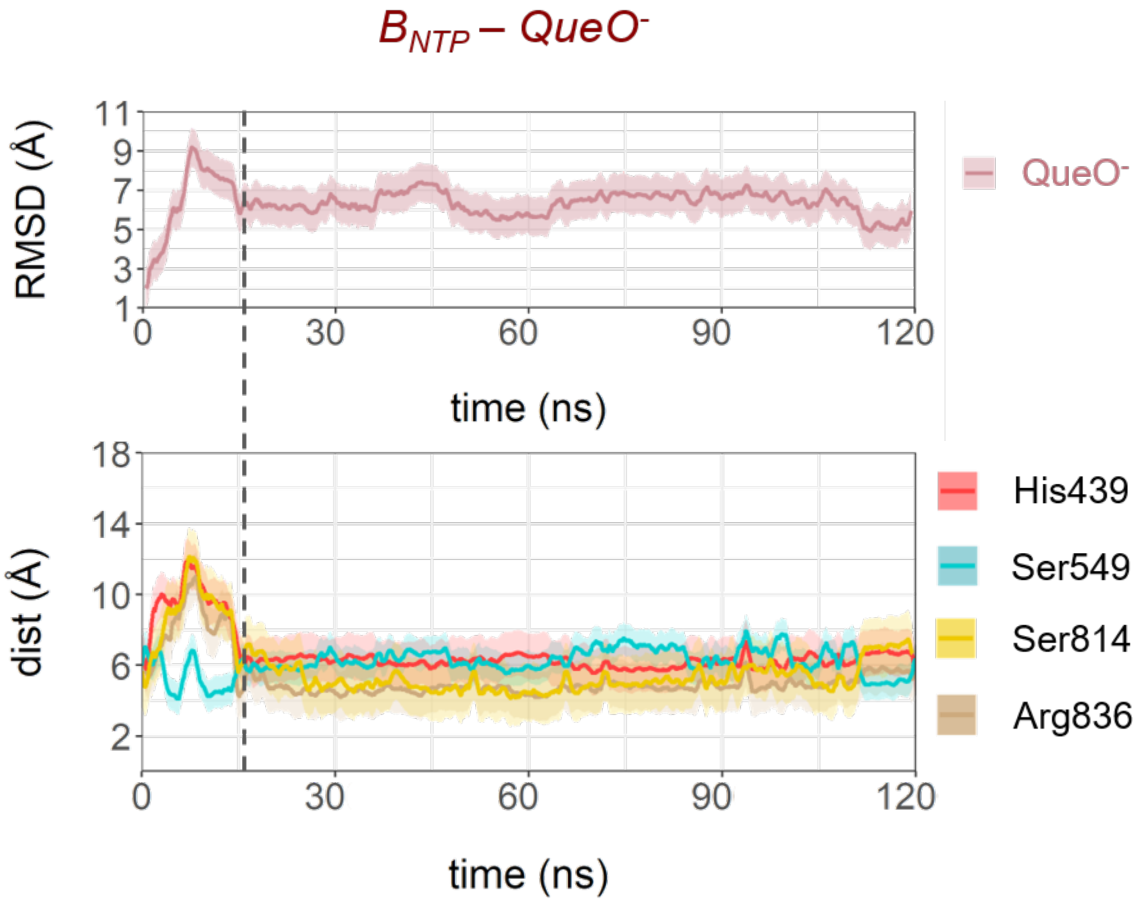

Figure S3. MD simulation of the B_{NTP}-$QueO^-$ system. (Top) Time evolution of the root mean square deviation (RMSD) for the heavy atoms of $QueO^-$. (Bottom) Time evolution of the distances between the center of mass (C.O.M.) of the heavy atoms of the $QueO^-$ ligand and i) the C.O.M. of the heavy atoms of the guanidinium group of Arg836 (in light brown), ii) the C.O.M. of the heavy atoms of the side chain of Ser549 (in pink), iii) the C.O.M. of the heavy atoms of the side chain of Ser814 (in cyan). The dashed line marks the reorganization of LutOH, and iv) the C.O.M. of the heavy atoms of imidazole group of His439. The dashed line marks the reorganization of LutOH.

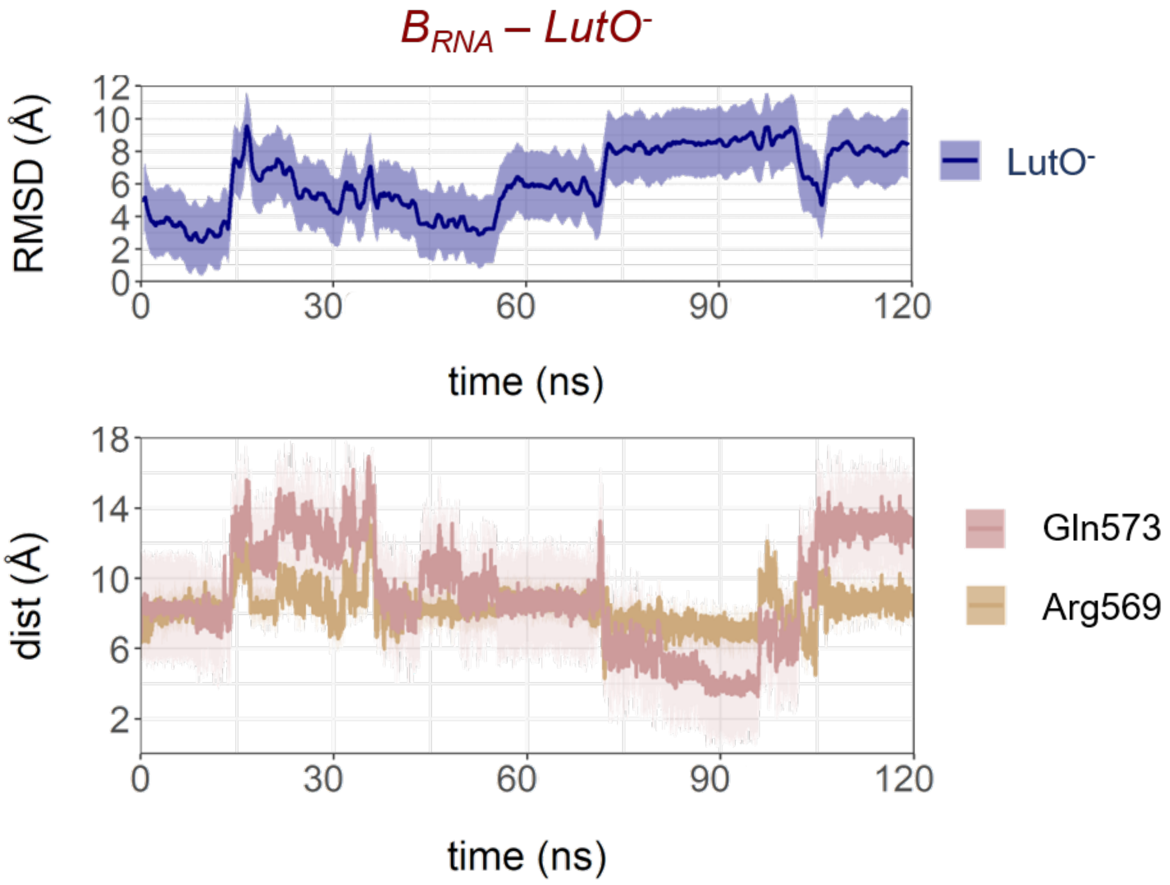

Figure S4. MD simulation of the B_{RNA}-$LutO^-$ system. (Top) Time evolution of the root mean square deviation (RMSD) for the heavy atoms of $LutO^-$. (Bottom) Time evolution of the distances between the center of mass (C.O.M.) of the heavy atoms of the $LutO^-$ ligand and i) the C.O.M. of the heavy atoms of the guanidinium group of Arg569 (in light brown), ii) the C.O.M. of the heavy atoms of the side chain of Gln573 (in pink).

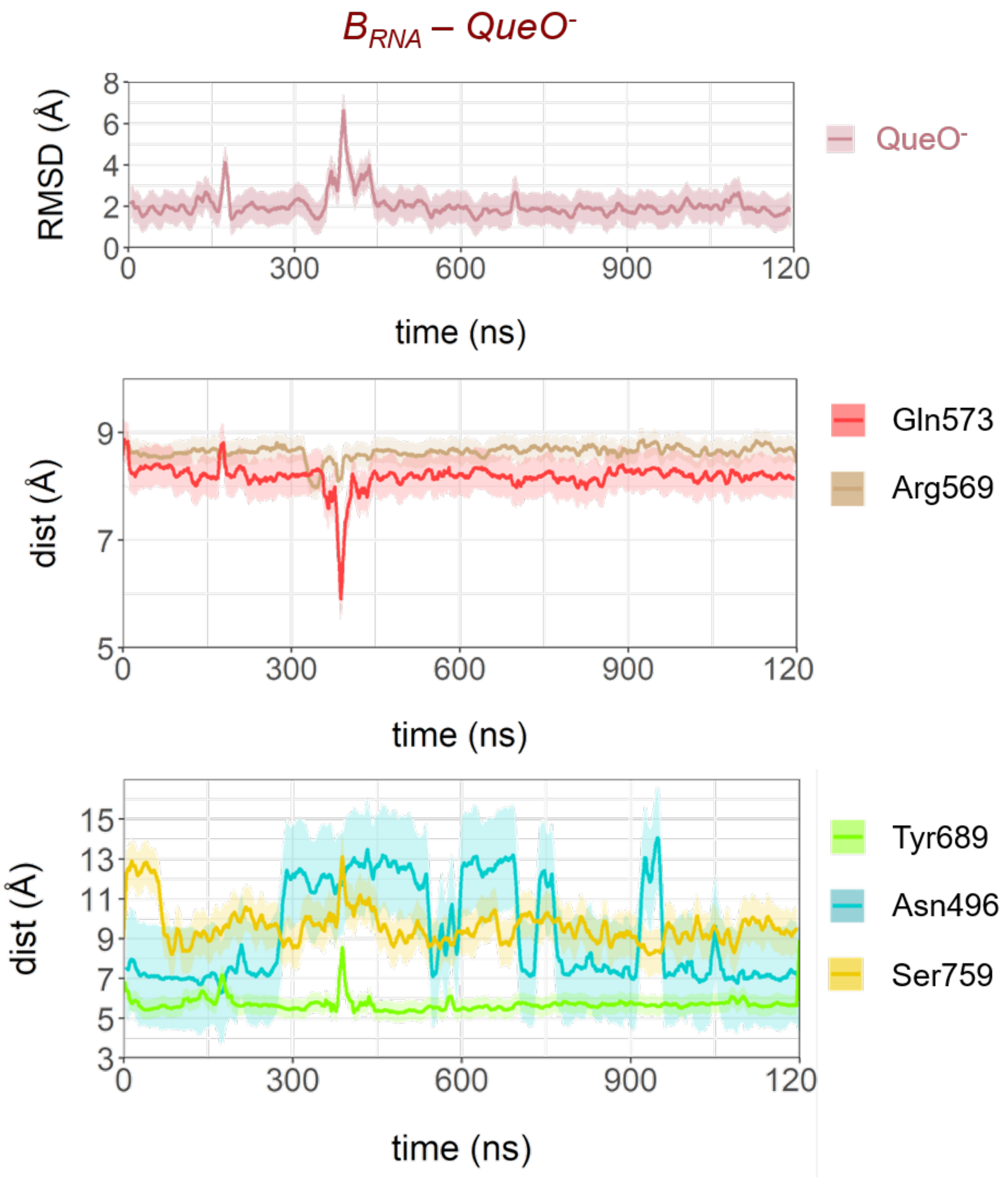

Figure S5. MD simulation of the B_{RNA}-$QueO^-$ system. (Top) Time evolution of the root mean square deviation (RMSD) for the heavy atoms of $QueO^-$. (Middle) Time evolution of the distances between the center of mass (C.O.M.) of the heavy atoms of the $QueO^-$ ligand and i) the C.O.M. of the heavy atoms of the guanidinium group of Arg569 (in light brown), ii) the C.O.M. of the heavy atoms of the side chain of Gln573 (in pink). (Bottom) Time evolution of the distances between the center of mass (C.O.M.) of the heavy atoms of the $QueO^-$ ligand and i) the C.O.M. of the heavy atoms of the side chain of Tyr689 (in green), ii) the C.O.M. of the heavy atoms of the side chain of Asn496 (in blue), and iii) the C.O.M. of the heavy atoms of the side chain of Ser759 (in yellow).
